# Rotavirus prevalence and seasonal distribution post vaccine introduction in Nairobi county Kenya

**DOI:** 10.11604/pamj.2019.33.269.18203

**Published:** 2019-07-29

**Authors:** Joshua Gikonyo, Betty Mbatia, Patrick Okanya, George Obiero, Carlene Sang, James Nyangao

**Affiliations:** 1 Department of Biochemistry and Biotechnology, School of Biological and Life Sciences, Technical University of Kenya, Nairobi, Kenya; 2 School of Pharmacy and Health Sciences, United States International University Africa, Nairobi, Kenya; 3 Kenya Medical Research Institute, Nairobi, Kenya

**Keywords:** Diarrhea, acute gastroenteritis, rotavirus, prevalence

## Abstract

Rotaviruses are one of the leading etiological agents of gastroenteritis in young children, for which a monovalent G1P(8) vaccine has been provided for free in Kenyan since July 2014. The main objective was to estimate the post vaccine prevalence and seasonal distribution of rotavirus diarrhea in children less than 5 years in Nairobi County, Kenya. Rotavirus positive samples were collected from children below 5 years of age in two hospitals within Nairobi County where vaccination status was card-confirmed. The children were examined and the demographic and clinical profiles of the children were recorded. Fecal specimens were analyzed for rotavirus antigen using an ELISA kit, followed by characterization by PAGE. Out of the total 323 samples, 49 had detectable rotavirus infection, representing 15.2% prevalence. Age distribution of rotavirus prevalence was as follows: ≤ 6 months-8.5%, 7-12 months-27.4%, 13-24 months - 41.4%, 25-36 months - 16.4% while 36-65 months had 6.3%. Rotavirus diarrhea was more common in wet and cold months of the year, the highest prevalence being observed in August (24.5%), 12.3% in both July and March, while April scored a prevalence of 10.2%. Out of the 49 rotavirus positive children, 48 had vomiting and abdominal cramps while all had fever and watery stool. The prevalence of Rotaviral diarrhea in children less than 5 years in Nairobi County Kenya has greatly reduced following the vaccine introduction and is more common during the wet and cold seasons of the year.

## Introduction

The World Health Organization (WHO) estimate that almost 2.5 billion episodes of diarrhea occur annually in children <5 years of age in developing countries [1]. More than 80% of these episodes occur in Africa and South Asia, 46% and 38%, respectively [2]. Rotavirus remains the leading etiology of severe and fatal diarrhea worldwide, responsible for approximately 40% of diarrheal deaths [3]. However, the introduction of rotavirus vaccines is believed to have a substantial impact on the burden of rotavirus diarrhea [4], with the majority of the impact seen by the third year after introduction [5]. Two oral live attenuated rotavirus vaccines (Rotarix and RotaTeq) have been licensed and introduced since 2006 [6]. Rotarix (GlaxoSmithKline) is a monovalent G1P [8] rotavirus vaccine (RV1) derived from a human rotavirus strain by serial passage in cell culture [7] while RotaTeq (Merck) is a pentavalent human-bovine (WC3) reassortant vaccine, with the five most common human type specificities G1, G2, G3, G4 and P [8], expressed individually on the genetic backbone of a bovine rotavirus strain naturally attenuated for humans [8]. The vaccine in use is administered orally to infants at 6 and 10 weeks of age globally, although different countries use different schedules [9]. As of May 1^st^ 2016, rotavirus vaccinations had been implemented in the national immunization programs of 81 countries, including 38 low-income countries being supported by the Global Alliance for Vaccines Initiative (GAVI) Alliance [10]. Before the vaccine introduction in Kenya, rotavirus group A (RVA) was estimated to cause more than 3,908 deaths, 3,015 outpatient visits, and 279 hospitalizations per 100,000 children <5 years of age annually. The cost to the health care system amounted to US$10.8 million annually [10]. With support from GAVI, Kenya introduced the two-dose RV1 into her national immunization programme in July 2014. The vaccine is administered orally at 6 and 10 weeks of age and the goal is to protect more than 1.5 million children in the country from developing severe acute gastroenteritis [10]. Since vaccine introduction, information on the actual impact of rotavirus vaccinations in preventing and reducing the health burden of severe childhood diarrhea in Kenya is limited. More so, an indication that the vaccines are much less efficacious in some low-income countries [11] underscores the importance of monitoring the impact of rotavirus vaccinations in low income settings during routine programmatic use, where the actual performance of a vaccine may differ from the optimal conditions of clinical trials. Hence, in this study, we report on the impact of rotavirus vaccination on the prevalence, age and seasonal distribution of rotavirus-specific gastroenteritis in urban population in Nairobi County, Kenya, three years after the introduction of the vaccine into the national immunization programme.

## Case study

**Study setup:** from January 2015 to December 2017, an active hospital-based surveillance for rotavirus gastroenteritis, in inpatients and outpatients of both Mbagathi and Mama Lucy district hospitals was conducted. The selected hospitals belong to different geographical regions within the County; Mbagathi District Hospital in Kibra Sub County and serves as the major referral hospital in the western part of the County. On the other hand, Mama Lucy District Hospital is in Embakasi West Sub County and serves as the major referral hospital in the eastern part of the County. Study subjects were infants and young children below the age of 5 years who had experienced an episode of three looser-than-normal or watery stools within a 24-h period for not more than 7 days, with or without episodes of vomiting [12]. The children either came directly from the community or were referred from peripheral health centers and dispensaries. Decisions on investigations, hospitalization and treatment were at the discretion of the clinicians attending to the children.

**Ethical considerations:** this study was approved by the Kenya Medical Research Institute (KEMRI) Scientific and Ethics Review Unit, (SSC NO. 015/3368). Written informed consent was obtained from the caregivers of all participating children after the nature and possible consequences of the study had been fully explained.

**Sample collection:** demographic and clinical data were collected from the children who met all of the inclusion criteria using a pathological investigation form adapted from the WHO generic protocol for rotavirus surveillance [13]. A team of experienced nurses were hired to recruit and consent study participants, as well as collect fecal samples in both Mbagathi and Mama Lucy District Hospitals. Children with acute gastroenteritis (AGE), defined as having ≥3 loose stools and/or ≥1 episode of unexplained vomiting followed by loose stool within a 24-hour period, abdominal pain and fever were asked to provide a stool sample for rotavirus testing. After obtaining written parental informed consent, fecal samples were collected in sterile stool caps from both outpatients and inpatients (children below five years of age). Each sample was labeled with the date of collection and a sample number was assigned. Vaccination status was card-confirmed. The specimens were first stored under -4^o^C at the hospitals before being transported to the Rotavirus Laboratory in KEMRI where they were stored at -20^o^C until processing.

**Virus detection:** the presence of RVA antigen in stool samples was determined using a commercially available EIA kit (ProSpecT^TM^ Rotavirus kit, Oxoid Ltd, UK) according to the manufacturer's instructions. Stool suspension (10%) was prepared in 1.5 ml labeled Eppendorf tube by adding 100µl stool sample to 1ml sample diluent from the kit, then vortexed for 1 minute to mix and left on the bench to settle. A 100µl of 10% stool suspensions were added in separate micro-wells, as well as the negative and the positive controls. Two drops (100µl) of enzyme conjugate were added in to the wells and incubated at 20-30^o^C for 60 minutes. The wells were washed with a wash buffer, after which the plate was inverted and tapped on absorbent paper to remove the traces of wash buffer. A hundred µl substrate 3,3',5,5'-tetramethylbenzidine (TMB) was added to each well and incubated in darkness at 20-30^o^C for 10 minutes. Two drops (100µl) of sulphuric acid were added to each well to stop the reaction. The results were read visually and then spectrophotometrically within 10-15 minutes at a wavelength 450 nm. The cut-off value was calculated by adding 0.200 absorbance units to the negative control value, which was then used as the standard of reference.

**Data analysis:** the rotavirus gastroenteritis prevalence, age and the seasonal distribution were analyzed using the Chi-Square (χ^2^) Test where a P-value of <0.05 was considered significant. We analyzed AGE due to rotavirus after vaccine introduction and used the pre vaccine data to calculate the percentage decline in the overall prevalence in rotavirus AGE and among the different age categories of children. However, we could not collect data on rotavirus vaccination status of each individual child due to challenges with record keeping. Data analysis was performed using the Graph pad prism version 5.0.

## Results

**Figure 1:**
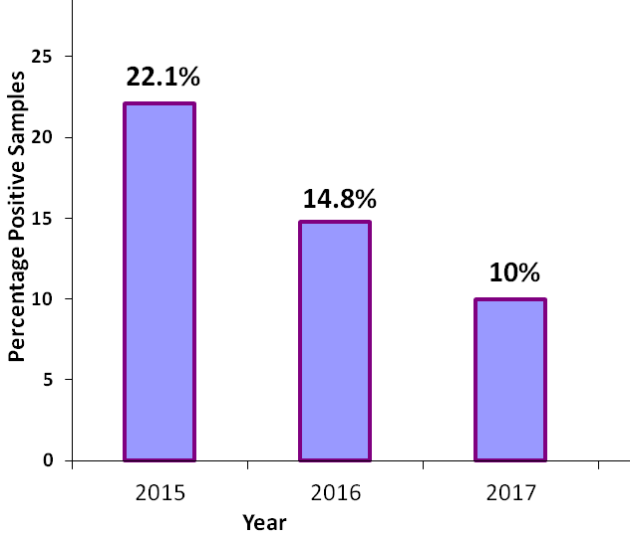
Yearly distribution of rotavirus infections in children aged five years and below in Nairobi county, between 2015 and 2017

**Prevalence of rotavirus gastroenteritis and proportional yearly distribution of rotavirus infections:**in each successive year beginning 2015 to 2017, rotavirus infection among children aged below five decreased significantly (Figure 1), and the yearly distribution was statistically different at P<0.05 (Table 1). Out of 323 fecal samples were screened for group A rotavirus using ELISA techniques, 49 had detectable rotavirus infection, representing a prevalence rate of 15.2%. In the year 2015, 21 out 95 (22.1%) samples collected were rotavirus positive. In 2016, 17 out 115 (14.8%) samples collected were rotavirus infected, while in the year 2017, 11 out of 113 (10%) samples tested rotavirus positive.

**Table 1:** Yearly distributions of rotavirus infections among children aged five years and below in Nairobi County

Year	Infected	Percentage	Uninfected	Percentage	Chi Squire	df	P value
**2015**	21	22.1	74	77.9			
**2016**	17	14.8	98	85.2			
**2017**	11	10	102	90	13.01	6	0.0428

**Demographic characteristics Gender distribution:** samples collected from female children were 165 out of a total of 323 (51%), while 158 (49%) were from male children. Of the 49 ELISA-positive specimens, 26 (53%) were from the female children, while 23 (47%) were isolated from the male counterparts. There was a close to equal distribution of rotavirus infections between the male and female children. With a P value of < 0.05 and 1 degree of freedom, the critical value was 0.09041 while the tabulated chi square value was 0.7637 (Table 2). Hence the viral infections were equally distributed between male and female children.

**Table 2:** Statistical gender distribution of rotavirus infections

Gender	Infected	Percentage	Uninfected	Percentage	Chi Squire	df	P value
Male	23	15	135	85	0.09041 1 0.7637		
Female	26	16	139	84			

**Figure 2:**
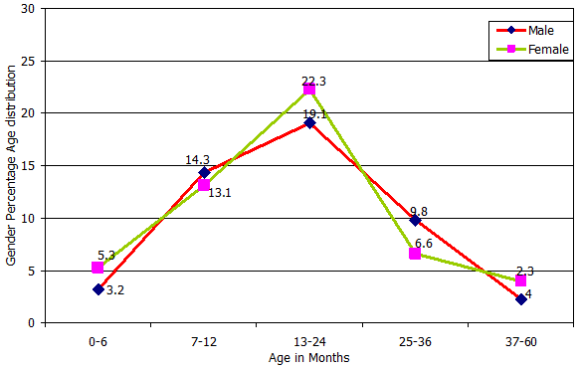
Proportional age distribution of rotavirus infection in male and female children below five years of age attending selected health facilities in Nairobi county, Kenya

**Age distribution of rotavirus infections:** as shown in Figure 2, the children aged 13 to 24 months had the highest infection (19% in males and 22% in females), while the least common rotavirus infections (4% in males and 2.3% in females) were observed among the 3 years and above age group. There was a reduction in the number of cases detected after two years of age, where only 11 samples were detected in the age group between 2 to 5. The age distribution of the children infected with rotavirus in the 3 years of study was statistically different. With P< 0.05 and 5 degrees of freedom; the critical value was 13.75 while the tabulated chi squire was 11.070. The calculated P value was 0.0173.

**Figure 3:**
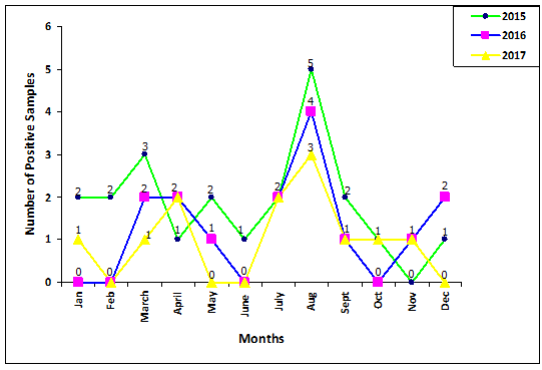
The yearly seasonal distribution of rotavirus infection in children below five years of age in Nairobi county

**Seasonal distribution of rotavirus infection:** the percentage infections observed in different seasons of the three years were as displayed in Figure 3. The highest incidences were reported in July-September in the three years followed by March-April period. However, despite the high peaks, the number of infections reduced over the three years with the number of positive infections in August 2015 being 10% and 6% in 2017. The seasonal distribution of rotavirus infection was significantly different (χ^2^ = 21.55; df = 11; P=0.0281) (Table 3).

**Table 3:** Seasonal distribution of rotavirus infections

Months	Infected	Percentage	Uninfected	Percentage	Chi Squire	dfd	P valuelue
**Jan**	3	18.8	13	81.2	21.55	11	0.0281
**Feb**	1	7.1	14	92.9			
**March**	6	10.4	52	89.6			
**April**	5	15.2	28	84.8			
**May**	3	18.8	13	81.2			
**June**	2	5.9	32	94.1			
**July**	6	12	44	88			
**August**	11	37.9	18	62.1			
**Sept**	5	33.3	10	66.7			
**Oct**	2	11.1	16	88.9			
**Nov**	2	12.5	14	87.5			
**Dec**	3	10.4	26	89.6			

**Clinical symptoms** the major clinical symptoms related to rotavirus infections were mainly watery stool/diarrhea, vomiting, abdominal cramps, and fever. All the children under study had watery diarrhea and fever, while 98% experienced vomiting and abdominal pains.

## Discussion

Reviews of rotavirus research conducted in Kenya before the vaccine introduction reveal rotavirus prevalence ranging from 6% to 56% in children aged below 5 years [14, 15]. In Nairobi County, a rotavirus prevalence of 24% was registered in children <5 years of age before the vaccine introduction [16]. The current study demonstrated substantial and continuous reductions in both rotavirus gastroenteritis hospitalizations and outpatient visits, in age cohorts eligible for vaccination in Nairobi County. In the year 2015, the study registered an infection rate of 22.1% (7.9% decrease), 14.8% (38.3% decrease) in year 2016, and 10% (58.3% decrease) in year 2017 (Figure 1). This was a total decline of 35%, where the reductions in rotavirus positivity rates were associated with a corresponding decline in the total number of children with positive results per year. Unlike in the pre vaccine era where the highest rate of infection was observed in the <12 months age bracket (vaccine eligible), the study registered the highest rate of infection in the 13-24 months age bracket. This corresponds to a study done on the peri-urban population in Central Kenya [10], where reductions in rotavirus positivity were most pronounced among the vaccine-eligible group (<12 months). The fact that the decline in the post-vaccine period was highest in children <1 year of age who were vaccine eligible, unlike in the pre-vaccine period where the under one year children experienced the highest rotavirus infection supports the idea that the reduction registered was associated with vaccine implementation. This is the first study to report on the rotavirus gastroenteritis status in Nairobi County, since the government vaccine introduction in 2014. The marked reductions in RVA-associated gastroenteritis observed in this study following the implementation of the rotavirus vaccine in Kenya reveal an overall prevalence of rotavirus infection, within the estimated ranges from previous studies done in Kenya and other parts of the world. Among the early introducing African countries, declines in RVA-associated AGE hospitalizations have been shown to range between 24% and 49% following the nationwide vaccine use [17]. The decline only occurred in children <1 year of age who were eligible for vaccination and was greatest during the rotavirus season months, supporting that it was associated with vaccine implementation [18]. In Kenya, a study conducted in the Western parts of the country revealed a 48% decline in the rotavirus related hospitalizations among children aged <5 years in the post-vaccine period [10].

In another study conducted in the peri-urban children of Central Kenya between 2014 and 2016 [17], an approximately 50% reduction in the rate of RVA gastroenteritis among children aged <5 years was observed, which was a slightly higher reduction compared to the 35% reduction registered in our study. This could have been due to the fact that our study captured low income urban setups of Nairobi County (including Kibra slums, Mathare slums, Dandora, Kayole and Mukuru slums) which are highly populated and where children experience poor environmental hygiene, and at the same time live on poor diet, unlike in the peri-urban setups of Central Kenya. Also, variation in rotavirus vaccine coverage by sub-counties in Kenya [19] may have contributed to the differences in the infection rates between these studies. Following the rotavirus vaccine introduction in Kenya, a marked reduction in RVA positivity occurred across all ages studied, with the highest decline being among infants <12 months of age, who include the vaccine-eligible population. Also, an increased decline in RVA-associated gastroenteritis among children aged 12-23 months during the second year of the vaccine introduction was observed. This reduction in rotavirus gastroenteritis was similar to that described in South Africa, Rwanda and Tanzania [3], and is consistent with reports from other African countries that were among the first countries to adopt the use of rotavirus vaccine [20-22]. The reports describe greater initial declines in the proportion of RVA cases among younger age groups that received vaccination in the initial years of the vaccination, followed by a progressive decline in older age groups in later years after its introduction. This is the herd effect of the rotavirus vaccine as described by Pitzer *et al*. 2009, and it provides further evidence that the declines can be attributed to the effect of rotavirus vaccination. Despite a clear and substantial impact of vaccine introduction, rotavirus remains the leading etiology of diarrhea in the third year after vaccine introduction. This highlights the critical importance of ongoing work to optimize the performance of rotavirus vaccination in these settings. A number of studies in many parts of the world have shown different rotavirus incidences, which is thought to be associated with geographical regions [23]. In the Kenyan pre-vaccine period, rotavirus diarrhea occurred throughout the year, but seasonal peaks were observed during the dry seasons of January-March and June-September [15]. In our three years of post vaccine study, a distinct seasonality of rotavirus infection was observed. There was a substantial decrease in the monthly counts of RVA infections among children <5 years of age compared to the pre-vaccine period. Whereas rotavirus infections in Kenya occurred year-round with seasonal peaks observed during the dry seasons in the pre-vaccine period, our three years post-vaccine study observed a peak of rotavirus infections in the months of August. However, rotavirus infections were recorded year-round in this study.

## Conclusion

The study registered substantial and continuous reductions in rotavirus gastroenteritis hospitalizations and outpatient visits in Nairobi County over a period of three years, following the July 2014 rotavirus vaccine introduction in Kenya. This decrease was most pronounced among the vaccine-eligible age group and was proportionate to the increasing vaccine coverage in Kenya. Thus, our findings suggest a significant public health impact of rotavirus vaccinations in Kenya. This study is among the first ones to give a report of the real-world impact of rotavirus vaccinations in Kenya since the introduction of the vaccine. Hence, our data provide early encouragement for stakeholders in the health sector in Kenya to support the sustained use of the rotavirus vaccine in the routine national immunization program. In addition, the demonstration of the herd effect of rotavirus vaccinations in this study is quite encouraging, since this has helped reduce the burden of rotavirus gastroenteritis in the non vaccine-eligible group of children.

## Competing interests

The authors declare no competing interests.
